# Factors associated with HPV-DNA clearance in a cohort of HIV-positive patients: role of cART and gender

**DOI:** 10.7448/IAS.17.4.19717

**Published:** 2014-11-02

**Authors:** Elisa Suardi, Francesca Bai, Laura Comi, Alessandro Pandolfo, Marco Rovati, Ambra Barco, Serena Dalzero, Barbara Cassani, Giulia Marchetti, Antonella D'Arminio Monforte

**Affiliations:** 1 Infectious Diseases, San Paolo Hospital/University of Milan, Milan, Italy; 2 General Surgery, San Paolo Hospital/University of Milan, Milan, Italy; 3 Gynaecology and Obstetrics, San Paolo Hospital/University of Milan, Milan, Italy; 4 Pathology, San Paolo Hospital/University of Milan, Milan, Italy

## Abstract

**Introduction:**

We aimed to assess any factors associated with dysplasia regression and with HPV clearance in a cohort of HIV+ patients, with particular focus on cART and gender.

**Methods:**

Asymptomatic HIV+ patients of the San Paolo Infectious Disease (SPID) cohort who underwent anoscopy/gynaecological evaluation were enrolled. Anal/cervical brushing were analyzed for: HPV-PCR detection/genotyping (HR-HPV), cytologic abnormalities (Bethesda System 2001: LSIL-HSIL). Demographics and HIV-related parameters were evaluated at baseline. Activated CD8+/CD38+ lymphocytes were measured (flow citometry). Patients were examined at baseline (T0) and at 12–18 months visit (T1). HPV clearance was defined as negativisation of HPV at T1; SIL regression (SIL-R) and progression (SIL-P) were defined as change from HSIL/LSIL to a lower-grade/absence of dysplasia and as change from absence of HSIL/LSIL to a higher-grade dysplasia at T1, respectively. Mann Whitney test, Chi-square test and multivariate logistic regression were used.

**Results:**

A total of 189 patients were examined, 60 (32%) were women. One hundred fifty patients (79%) were HPV+, 113 (75%) harboured HR-HPV; 103 (68%) showed LSIL/HSIL at T0 (32% of women and 65% of men) (all were HPV-positive). No differences in demographics and HIV-related markers were found between patients with SIL-P (33, 41%) and patients with SIL-R (47, 59%). HPV+ patients who cleared HPV (28, 18%) were found to be more frequently female, heterosexual infected, more frequently on cART and with lower Log10 HIV-RNA and lower levels of CD8+/CD38+ % compared with HPV persistence group (Table 1).

No differences in PI exposure were found between the two groups (p=.08). Interestingly, also when only HR-HPV were considered, clearance was associated with exposure to cART (naïve 4%, vs cART 86%, p=.048). In multivariate analysis, heterosexuals (AOR 5.123, 95% CI 1.5–17.5 vs homosexuals) were independently associated to HPV clearance, whereas CD8+/CD38+% (AOR 0.44, 95% CI 0.65–1.01 for each % more) were predictive of HPV persistence.

**Conclusions:**

Close follow-up of HPV and SIL should be promoted particularly in men and in untreated individuals. We cannot exclude behavioural variables linked to risky sex and reinfection.

**Table 1:** Characteristics of study population according to HPV clearance

Characteristics of Study Population	Patients that cleared HPV (n=28)	Patients that remain HPV+ (n=122)	p
Female Sex	11 (39%)	19 (15%)	0.05
Heterosexual epidemiology	16 (57%)	29 (23%)	0.001
Log10 HIV-RNA*	1.77 (1.59–1.77)	1.77 (1.59–4.16)	0.038
cART	28 (100%)	122 (81%)	0.015
CD8+/CD38+%*	1 (1–2.7)	2 (1–6)	0.001

